# Amplification of neural stem cell proliferation by intermediate progenitor cells in *Drosophila *brain development

**DOI:** 10.1186/1749-8104-3-5

**Published:** 2008-02-19

**Authors:** Bruno C Bello, Natalya Izergina, Emmanuel Caussinus, Heinrich Reichert

**Affiliations:** 1 Biozentrum, University of Basel, CH-4056 Basel, Switzerland

## Abstract

**Background:**

In the mammalian brain, neural stem cells divide asymmetrically and often amplify the number of progeny they generate via symmetrically dividing intermediate progenitors. Here we investigate whether specific neural stem cell-like neuroblasts in the brain of *Drosophila *might also amplify neuronal proliferation by generating symmetrically dividing intermediate progenitors.

**Results:**

Cell lineage-tracing and genetic marker analysis show that remarkably large neuroblast lineages exist in the dorsomedial larval brain of *Drosophila*. These lineages are generated by brain neuroblasts that divide asymmetrically to self renew but, unlike other brain neuroblasts, do not segregate the differentiating cell fate determinant Prospero to their smaller daughter cells. These daughter cells continue to express neuroblast-specific molecular markers and divide repeatedly to produce neural progeny, demonstrating that they are proliferating intermediate progenitors. The proliferative divisions of these intermediate progenitors have novel cellular and molecular features; they are morphologically symmetrical, but molecularly asymmetrical in that key differentiating cell fate determinants are segregated into only one of the two daughter cells.

**Conclusion:**

Our findings provide cellular and molecular evidence for a new mode of neurogenesis in the larval brain of *Drosophila *that involves the amplification of neuroblast proliferation through intermediate progenitors. This type of neurogenesis bears remarkable similarities to neurogenesis in the mammalian brain, where neural stem cells as primary progenitors amplify the number of progeny they generate through generation of secondary progenitors. This suggests that key aspects of neural stem cell biology might be conserved in brain development of insects and mammals.

## Background

Neural stem cells are primary precursors that have the ability to renew themselves at each division such that one of the two daughter cells retains stem cell identity, while the other enters a program of differentiation and contributes to a continuous supply of neural cell types. Understanding how neural stem cells maintain their pluripotent state and how their progeny differentiate into distinct neural fates is of central importance for understanding nervous system development (for recent reviews, see [1-3]). Neural stem cells must exert a tight control over proliferative divisions so as to generate the appropriate number of neural progeny necessary to populate the nervous system but not to produce so many self-renewing daughters that neoplastic overgrowth occurs [4]. Therefore, a better comprehension of the mechanisms that control the behavior of neuronal stem cells and their progeny may also be important for understanding brain tumors [5,6].

The *Drosophila *central nervous system is an excellent simple model system for analyzing the molecular mechanisms that control neural stem cell divisions (for recent reviews, see [7,8]). *Drosophila *neural stem cells, called neuroblasts (NBs), delaminate as single cells from the neuroectoderm and undergo repeated asymmetric cell divisions, each of which self-renew the NB while producing a smaller neural progenitor cell called a ganglion mother cell (GMC). Compared to the NB, the GMC adopts a radically opposite fate and undergoes a single neurogenic division to produce two cells that exit the cell cycle and differentiate (reviewed in [9-12]). During embryogenesis, each NB produces a lineage of 10–20 primary neural cells that contribute to the functional circuitry of the larva. Following a period of quiescence, most NBs resume their asymmetric mode of proliferative divisions during post-embryonic development and generate the lineage-related clusters of secondary adult-specific neurons that make up the bulk of the adult central brain and thoracic ganglia [13-16].

Mechanisms involved in NB division and neural proliferation during embryogenesis have been studied in great detail (reviewed in [7,17-19]). NB divisions are known to be molecularly as well as morphologically asymmetric, and a number of key intrinsic and extrinsic factors that control the asymmetrical and self-renewing divisions of these NBs have been identified. Among these, a central role is played by molecular polarity cues that establish the apico-basal polarity of the NB and enable the asymmetric segregation of localized cell-fate determinants from the NB to the GMCs at each asymmetric cell division. Although considerable insight has been attained into the mechanisms by which NB polarity is established and maintained, little is known about the function of the proteins that are asymmetrically localized to the GMC. The best characterized of these fate determinants is the homeodomain protein Prospero, which is synthesized in the NB and localized at the cell cortex in a polarized manner. Upon segregation to the GMC, Prospero acts in the nucleus to repress NB-specific gene expression (including genes required for self-renewal) and activate genes for GMC fate specification and terminal differentiation of post-mitotic neurons [20-23]. Asymmetric segregation of Prospero protein is mediated by the adaptor coiled-coil protein Miranda. Once segregated from the NB to the GMC, Miranda is degraded, thereby releasing Prospero from the cell cortex and allowing it to enter the nucleus [24-26]. Indeed, the nuclear localization of Prospero is one of the first molecular differences between the self-renewing NB and a differentiating cell [27,28].

During the postembryonic period of neurogenesis, the NBs of the central brain and thoracic ganglia are thought to undergo a similar proliferation program and express many of the asymmetric cell fate determinants that characterize embryonic neurogenesis [29,30]. Nuclear localization of Prospero is manifest in GMCs and postmitotic neurons of the larval brain, and loss of *prospero *in somatic clones results in massive overproliferation of cells that express molecular markers of NBs [31-33]. Additionally, numerous other molecular control elements are likely to be required for the continuous mitotic activity of NBs during postembryonic life (reviewed in [34]).

Controlled neuronal proliferation is especially important for the generation of the adult brain. The mature brain of *Drosophila *is an exceedingly complex structure with numerous highly organized neuropil assemblies, such as the mushroom bodies, central complex and antennal lobes, as well as other specialized neuropils and major fiber tracts required for complex behavioral functions [35]. Remarkably, approximately 95% of the neurons that make up the adult brain are post-embryonic in origin, and in the central brain all of these neurons are produced by a set of only about 100 bilaterally symmetrical NBs [36,37]. Given the fact that 100 NB pairs generate the tens of thousands of differentiated, spatially heterogeneous neurons in the adult central brain, sophisticated mechanisms for lineage- and region-specific amplification control of NB proliferation are likely to be required during post-embryonic brain development. However, with the exception of rough estimates, which suggest that each brain NB might undergo between 40 and 60 rounds of post-embryonic mitosis to produce lineages of 100–150 neurons, very little is known about this process and the underlying molecular mechanisms.

Here we report that a striking amplification of neuronal proliferation is achieved by specific brain NBs during postembryonic development through the generation of intermediate progenitor cells (IPs). Using cell lineage-tracing and marker analysis, we show that remarkably large NB lineages develop in the dorsomedial (DM) area of the larval brain. Like any other lineages in the brain, they derive from unique NB precursors that remain associated with their post-mitotic neuronal progeny. In addition, they contain a large pool of cells that do not express neuronal differentiation markers, are engaged in the cell cycle, and show mitotic activity. While some of these mitotically active cells are GMCs, the others express NB-specific molecular markers and divide repeatedly to produce neural progeny, implying that they are IPs. The proliferative divisions of these IPs are morphologically symmetrical, but molecularly asymmetrical in that cell fate determinants such as Prospero and Miranda are segregated into only one of the daughter cells. The IPs are generated by a specific set of NBs that do not segregate Prospero to their smaller daughter cell, thereby allowing this cell to retain proliferative capacity instead of undergoing its final neurogenic division. The amplification of NB proliferation through IPs reported here for *Drosophila *bears remarkable similarities to mammalian neurogenesis, where neural stem cells as primary progenitors often amplify the number of progeny they generate via symmetrically dividing secondary progenitors (reviewed in [2]). This suggests that key aspects of neural stem cell biology might be conserved in brain development of flies and mammals.

## Results

### Large neuroblast lineages are located in the dorsomedial brain hemispheres

**Figure 1:**
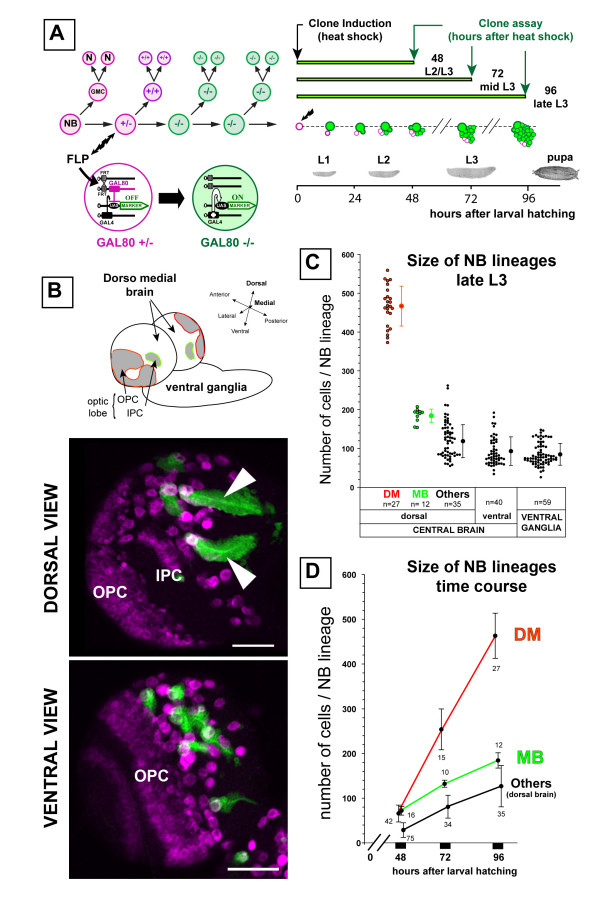
**The DM brain NBs generate a large number of progeny during larval development.(a) **Lineage labeling of a NB by MARCM. Left: schematic representation of a NB lineage in transgenic flies carrying a repressor transgene GAL80 distal to an FRT site in heterozygous (±) conditions. Ubiquitous expression of GAL80 under tubulin promoter control (pink) prevents GAL4-driven expression of the mCD8::GFP marker gene (green). Heat shock-induced FLP recombinase (FLP) at a given time point mediates the FRT site-specific mitotic recombination. Segregation of recombinant chromosomes at mitosis may result in the loss of the GAL80 repressor transgene in the NB daughter, which allows stable expression of the marker in this cell and its progeny. After several rounds of division such a positively labeled clone contains the NB, one or more GMCs and numerous post-mitotic neurons (N). Right: following random heat-shock induced NB recombination in newly hatched larvae, the size and composition of isolated NB lineages were examined at different time points during larval development. **(b) **NB clones were examined in all parts of the brain and ventral ganglia with the exception of optic lobes. The latter are easily recognizable in a single brain hemisphere by their lateral position and the high density of cells that express the progenitor marker Miranda (magenta, lower panels). On confocal images of brain hemispheres at low magnification (lower panels), GFP-labeled NB clones are easily identifiable by the presence of a large Miranda-positive NB and an associated cluster of clonal progeny. Unusually large clones could be identified in the dorsomedial part of the brain hemispheres (arrowheads). Anterior is to the top and lateral is to the left for each view. OPC and IPC, outer and inner proliferating centers, respectively. Scale bars: 50 μm. **(c) **The size of NB lineages was determined by counting cells in isolated clones plotted on the diagram according to their position in the nervous system (x axis). Each dot represents a clone with the mean ± standard deviation indicated by dots and error bars next to each group. DM, dorsomedial NB lineage; MB, mushroom body NB lineage; n, number of clones examined in each area. **(d) **Growth rate of different lineages examined at different time points after clone induction. Dots and bars represent the average size and standard deviation determined from the indicated number of clones.

Since most of the secondary, adult-specific neurons of the brain are generated during larval development [38], we used mosaic-based MARCM techniques to label NB lineages (hereafter referred to as 'NB lineages' or 'NB clones') in the developing larval nervous system [39]. Random mitotic recombination was induced in NBs within a few hours after larval hatching (ALH) in order to achieve positive labeling of their clonal post-mitotic progeny (Figure 1a). Labeled NB clones typically consisted of a single NB, unequivocally recognizable as a large cell of roughly 10 μm in diameter, and an associated cluster of smaller cells representing its larval progeny (Figure 1a,b) [40,41].

Prominent among these were unusually large clones recoverable at the DM margins of the brain hemispheres (Figure 1b). Six NBs located in the most medial position of each hemisphere were found to generate this type of clone, hereafter referred to as 'DM lineages' or 'DM clones'. As detailed below, the parental DM NBs were easily identifiable owing to the signature pattern of Miranda-positive cells that followed the lateral to medial orientation of their progeny in these labeled clones. Morphologically, DM NBs were indistinguishable from other NBs in the central brain or in the ventral ganglia. Thus, cell volume measurements of DM and non-DM NBs in third larval instar brains gave comparable values of 344 ± 94 μm^3 ^(n = 12) and 424 ± 110 μm^3 ^(n = 13), respectively. Preliminary analysis of the axonal tracts suggests that the large NB clones in the dorsal brain correspond to the pl and pm subgroups of the Dorsoposterior medial (DPM) lineages previously described (data not shown) [16].

To compare the proliferative capacity of the DM NBs with that of other NBs in the larval central nervous system, we quantified the number of cells in DM NB lineages, in mushroom body NB lineages, and in other NB lineages scored randomly in different brain and ventral ganglion regions of the late third instar larvae shortly before pupation (96 h ALH). The number of cells in the DM lineages had an average value of 450 (range 370–580). Remarkably, this was more than twice the average number of cells observed for the larval lineages of the mushroom body NBs (184 ± 17, n = 17) or for other larval NB lineages scored in other areas of the central nervous system (Figure 1c).

To determine the rate of clone size increase during larval central nervous system development, we counted the number of cells in MARCM-labeled DM NB clones, mushroom body NB clones and other dorsal brain NB clones at various larval stages (Figure 1d). Following a quiescent phase in the early developing larva, most NBs had entered mitosis by the late second larval instar stage [38]. Our observations show that at this stage (48 h ALH), NBs in the dorsal brain had generated only a small number of postembryonic cells and that no pronounced lineage-specific differences in progeny number was apparent (Figure 1d, 48 h ALH). However, at 72 h and 96 h ALH, the DM lineages had increased markedly in size when compared to other dorsal brain NB lineages, indicating an approximate four-fold increase in their rate of proliferation (Figure 1d).

To investigate this further, we cultured MARCM-labeled brain explants in 5-bromodeoxyuridine (BrdU) and then used anti-BrdU immunocytochemistry to determine the number of cells engaged in S-phase in DM clones compared to other NB clones of the central brain. Following a 90 minute pulse of BrdU incorporation in L3 brain explants, we found a markedly higher number of BrdU-positive cells in DM clones (38 ± 8 BrdU positive cells, n = 8 clones) than in the other NB clones scored at random in dorsal brain regions of the same specimens (4 ± 1.5, n = 27). (This higher rate of BrdU incorporation in DM clones was also observed at earlier stages and in various conditions of incubation; data not shown.)

These data indicate that a significant amplification of proliferation occurs in the DM lineages when compared to other NB lineages of the central brain (hereafter collectively referred to as 'non-DM' lineages).

### DM lineages contain a large population of mitotically active progenitor cells

The large number of cells found in the DM NB clones could, in principle, be due to an unusually high rate of mitotic activity of the DM NBs. However, immunodetection of mitotic DNA in MARCM clones (via the phospho-histone H3 (PH3) epitope) revealed a comparable mitotic frequency in these NBs (22.5%, n = 40) compared to NBs found in dorsal (16.7 %, n = 48) or ventral (21.6 %, n = 97) brain lineages. This prompted us to search for other types of progenitor cells in these lineages. To this aim, we first characterized molecular markers enabling *in situ *detection of mitotically active versus post-mitotic cells in labeled NB lineages of the larval brain.

**Figure 2:**
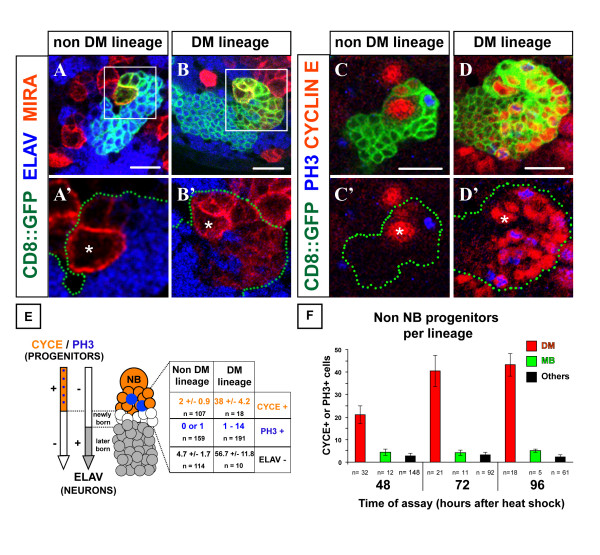
**The DM NBs generate an exceptional number of neuronal progenitors.(a-d') **Confocal images of representative non-DM and DM lineages labeled with mCD8::GFP (membrane marker, green) in larval brains stained for the markers indicated. Each panel shows the most superficial area of a single NB clone viewed around the NB (asterisk) in the dorsal brain. The GFP channel is omitted for clarity in the lower panels and green dots outline the clones. Note that (a', b') show close up views of the areas boxed in (a, b). Progenitor cells in an NB lineage include the NB identifiable by its size (asterisk) and the most recently born cells in its associated progeny. These cells are found in close spatial proximity to the NB and are characterized by a weak level of cortical Miranda (red in a-b') and the absence of the neuronal marker ELAV (blue in a-b'). **(c-d') **NB-associated cells are unambiguously defined as progenitors by the expression of the cell cycle markers Cyclin E and/or PH3. **(e) **Quantification of various markers in NB clones at 96 h ALH underscores the high number of small progenitor cells among the progeny of the DM NBs. **(f) **DM NBs are always associated with the highest number of non-NB progenitors during larval development. Cell counts were performed on three types of clones recorded on the same samples for comparisons: DM, dorsomedial NB clones; MB, mushroom body NB clones; others, clones chosen at random in dorsal areas of the brain and not belonging by position and morphology to the other groups. In each case, the average number of progenitors is plotted with an error bar representing standard deviation. The number of clones examined is indicated bellow. Scale bars: 10 μm.

Typically, in all NB clones examined, the majority of the labeled cells expressed the neuronal identity marker Elav. Prominent exceptions were the large NBs and a set of smaller cells closely associated with the NBs, all of which were Elav-negative (Figure 2a,b,a',b'). Quantification of the number of these Elav-negative cells revealed a striking difference in DM lineages compared to non-DM lineages (Figure 2e). DM lineages contained an average of 56.7 ± 11.8 Elav-negative cells (n = 10 clones) closely associated with the Elav-negative NBs. This was over 10 times more than in non-DM NB clones (4.7 ± 1.7 cells, n = 114), suggesting that the DM lineages contain a markedly higher number of mitotically active progenitor cells.

Could these smaller Elav-negative cells associated with the NBs be GMCs? To investigate this, we first studied the expression of the coiled-coil protein Miranda. The *miranda *gene has been reported to be expressed in larval NBs but not in their GMCs [42]; Miranda expression might, therefore, be a useful marker for differentiating NB-like cells from GMCs. In non-DM lineages, Miranda was strongly expressed in the NBs but only very weakly expressed in the set of smaller, Elav-negative cells associated with the NBs, suggesting that these Elav-negative cells were GMCs (Figure 2a,a'). (Their weak expression of Miranda could be due to perdurance of the protein during cell divisions; see also [29,30]). In DM lineages, Miranda was strongly expressed in the NB; however, in contrast to non-DM lineages, distinct Miranda expression was also observed in many of the smaller, Elav-negative cells associated with the NBs (Figure 2b,b'). This suggests that the smaller Elav-negative/Miranda-positive cells in the DM lineages might not be GMC-like, but might have properties that are more NB-like. To investigate this further, we next attempted to find other markers for progenitor cells and, thus, examined the expression of Cyclin E (CycE) and PH3 as markers of mitotically active cells.

In green fluorescent protein (GFP)-labeled non-DM NB clones, used as control, a small number of GMCs were observed as small NB-associated cells expressing either CycE or PH3 (Figure 2c,c'). At 96 h ALH we found an average of two CycE-positive cells (range one to five) and a maximum of one cell engaged in mitosis as visualized by anti-PH3 (Figure 2e) [40]. This pattern was consistent with live imaging data obtained in experiments on cultured nervous systems to monitor asymmetric NB divisions [43]. Thus, as in the embryo, these larval NBs divide by a budding process that generates a set of smaller GMCs, each GMC is born adjacent to the previous one, and the division of the 'oldest' GMC is delayed compared to that of the NB.

Contrasting with this simple pattern, DM lineages contained an average of 38 CycE-positive cells located around the NB, and many scattered mitoses, up to 14 per clone, were observed by PH3 immunoreactivity (Figure 2d,d',e). This strikingly high level of ongoing mitotic activity and engagement in the cell cycle in DM lineages compared to other central brain lineages (including mushroom body lineages) was seen at all stages of larval development examined (Figure 2f). These findings indicate that significantly elevated mitotic activity occurs among the numerous small NB-associated cells in larval DM lineages. Moreover, they are in accordance with the idea that these cells do not adopt a GMC fate, but rather remain mitotically active and continue to proliferate. In this case, these cells would have the characteristics of IPs that amplify the proliferation of their parent NBs (primary progenitors) in the DM lineages.

### Molecular markers reveal two types of non-neuroblast progenitor cells in DM lineages

If some of the mitotically active cells in DM NB clones are amplifying IPs, they might be expected to have cellular and molecular features in common with proliferating NBs. To investigate this, we first examined the expression patterns of Prospero, Miranda, and CycE in NBs of non-DM lineages, used as control, as well as in the small NB-associated progenitors of the DM lineages. For this, MARCM clones induced at larval hatching were scored at 96 h ALH. Importantly, we further restricted our analysis to cells engaged in mitosis (PH3-positive) in order to identify progenitor cells unambiguously and to obtain valid comparisons, since all markers showed cell-cycle dependent expression (see below). (Clones analyzed at 48 h or 72 h ALH gave comparable results; data not shown.)

**Figure 3:**
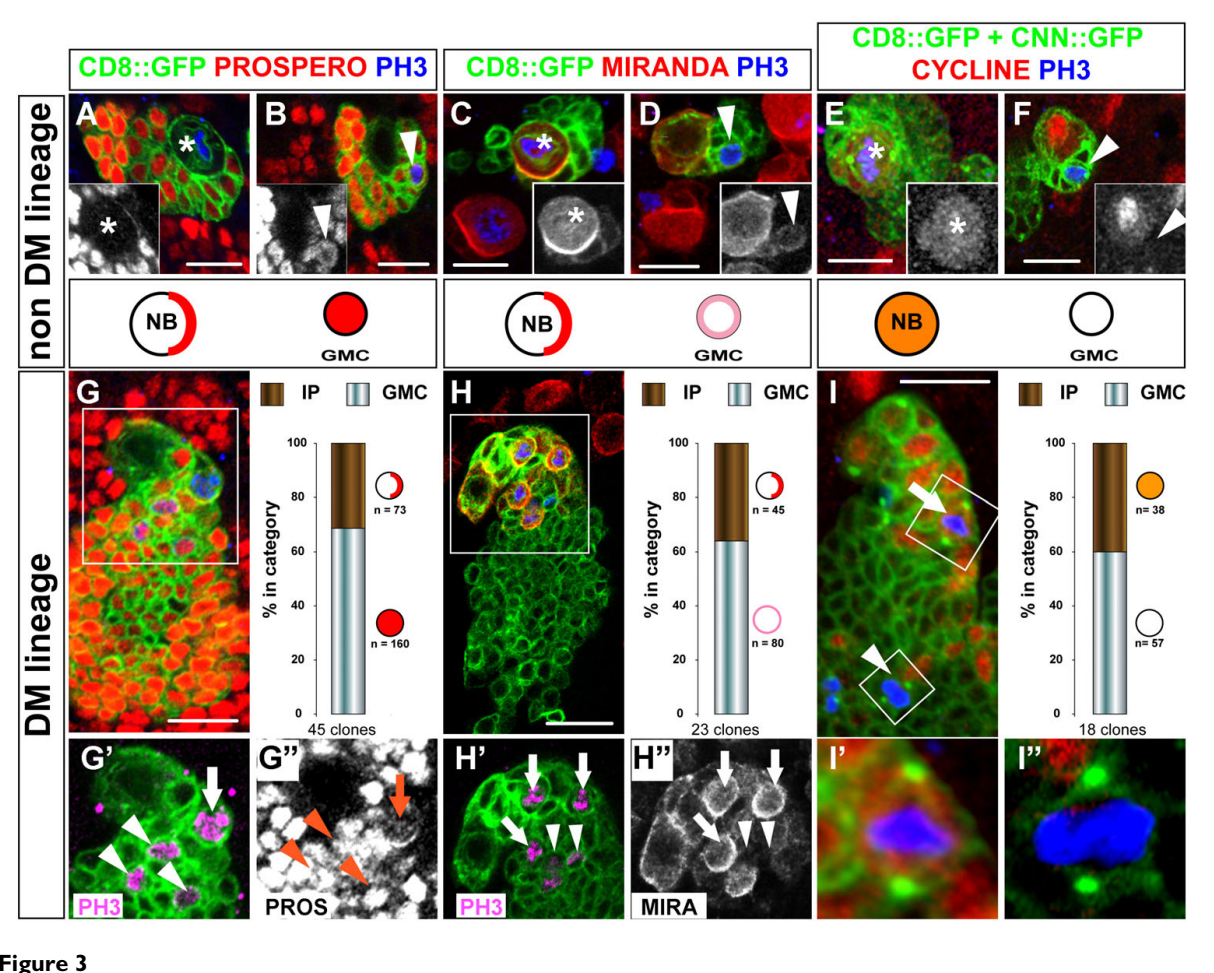
**Molecular characterization of NB-like and GMC-like progenitors in the progeny of DM NBs.** Confocal images of MARCM-labeled NB clones in the dorsal part of larval brains stained for the markers indicated on the top of the columns. Representative views of **(a-f) **non-DM lineages are used as a reference for **(g-i") **the DM lineages. Clones were labeled with CD8::GFP (membrane marker, green in all panels) and CNN::GFP (centrosomes visualized as bright green spots in e, f, i-i"). Proliferative cells are detected by anti-Cyclin (red in e, f, i-i') and anti-PH3 during mitosis (blue in all panels). In a non-DM NB clone, mitosis is restricted to two cell types: the NB and a single GMC in close proximity (a-f, asterisks and arrowheads, respectively). NBs show a unique pattern of polarized expression of Prospero and Miranda at the cell cortex during mitosis (a, c) and stable expression of Cyclin E throughout the cell cycle (e, mitosis; f, interphase). In contrast, the GMC is uniquely defined when engaged in mitosis (PH3 positive) by nuclear localization of Prospero (b, inset), weak uniform cortical localization of Miranda (d, inset) and lack of Cyclin E (f, inset). (g-i) In DM clones many progenitors other than the NB are identified as PH3-positive nuclei. These cells show patterns of marker expression usually found in mitotic NBs (IP; arrows) or mitotic GMCs (arrowheads). Lower panels show close up views of the areas boxed in (g-i). The two types of mitotic progenitors can be detected simultaneously in a single DM lineage (images) and are found at a comparable ratio when quantified in multiple clones using the three independent markers (histograms). IP, small NB-associated intermediate progenitor with NB-like marker expression. Scale bars: 10 μm (a-f) or 15 μm (g-i).

In non-DM clones, Prospero was specifically detected at the cellular cortex of the NBs, accumulating on one side during mitosis (Figure 3a; n = 57; 100%). All other cells in the clones expressed Prospero in the nucleus or uniformly throughout the cell, thus including both GMCs and post-mitotic cells. Localization of Prospero was more specifically revealed in the GMCs by co-staining with anti-PH3 (Figure 3b; n = 37; 100%) or CycE (not shown). In striking contrast, in DM lineages 31% of PH3-positive small NB-associated cells expressed Prospero at the cortex in a polarized manner. This expression pattern was, thus, similar to that observed in dividing NBs (Figure 3g,g", arrow). The remaining dividing, NB-associated cells showed uniform expression of Prospero throughout the cell at mitosis; their pattern was, thus, GMC-like (Figure 3g,g' arrowheads).

As expected, the adaptor protein Miranda formed prominent cortical crescents in dividing NBs of non-DM clones (Figure 3c, asterisks). In the associated GMCs, Miranda was detected at weaker levels with uniform cortical distribution both at interphase and during mitosis (Figure 3c, inset, and Figure 3d, arrowheads). Strikingly, in DM lineages, 36% of the NB-associated cells showed strong and polarized expression of Miranda during mitosis, as described for dividing NBs (Figure 3h,h", arrows). The remaining dividing cells showed weak and uniform cortical localization of Miranda; their Miranda expression pattern was, thus, GMC-like (Figure 3h,h' arrowheads).

To confirm the presence of both NB-like and GMC-like progenitors in the DM NB lineages, we searched for markers of cellular identity that did not rely on the conventional criteria of cell size and/or cortical polarity. Significantly, we found that in non-DM lineages (taken as reference lineages), CycE was detected in virtually all the self-renewing NBs during mitosis (Figure 3e, asterisks; n = 74), but never during the terminal division of the GMCs (Figure 3f, arrowheads; n = 48). This distinctive criterion for cell identity was only applicable during mitosis because all progenitor cells expressed CycE at interphase, irrespective of their size (Figure 3e,f; PH3^- ^nuclei; see also Figure 2c,d). In DM lineages, some of the small PH3-positive cells were negative for CycE but other small PH3-positive cells were positive for CycE (Figure 3i,i', arrow and arrowhead). Thus, in agreement with the data obtained using markers of cell polarity, both NB-like and GMC-like progenitors could be identified simultaneously in the progeny of a single DM NB (Figure 3g–i). Furthermore these two types of progenitors were observed specifically in these lineages and at all larval stages examined. Thus, the small CycE-positive/PH3-positive progenitors represented 55% (n = 64), 45% (n = 93) and 40% (n = 105) of the mitotic cells found in DM NB clones at 48 h ALH, 72 h ALH and 96 h ALH, respectively. The small CycE-positive/PH3-positive progenitors were never found associated with NBs of the ventral brain or the ventral ganglia at the corresponding stages (114 PH3-positive cells in 297 clones examined).

Taken together, these data indicate that the larval DM lineages contain two types of molecularly distinct progenitor cells other than NBs. Although not readily identifiable by their size, approximately two-thirds of these cells have molecular expression patterns of Prospero, Miranda and CycE that are characteristic of GMCs. In contrast, the remaining third have expression patterns of Prospero, Miranda and CycE that are remarkably similar to the patterns found in proliferative NBs. These novel NB-like progenitors are hereafter referred to as IPs. Our data further show that IPs are generated by DM NBs throughout larval neurogenesis in a quantitatively stable and balanced ratio with GMC-like progenitors and post-mitotic neurons.

### Intermediate progenitor cells divide repeatedly and produce multicellular neuronal clones

**Figure 4:**
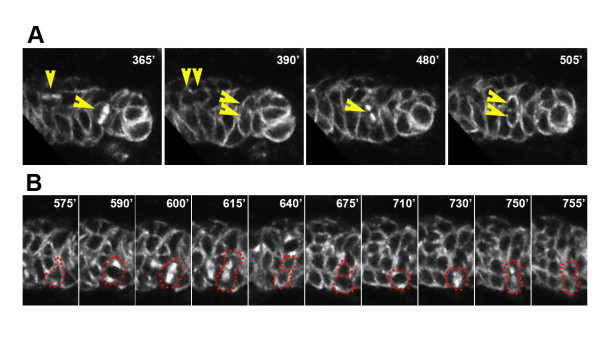
**Live imaging of multiple and repeated division of DM NB daughter cells in MARCM-labeled clones.** Frames from time-lapse recordings of a DM clone labeled with CD8::GFP and tau::GFP in larval brain cultured over 13 hours. The large NB, not visible in these frames, divided twice during this time period (Additional data file 1). The time is indicated in minutes relative to the start of the recording. **(a) **Multiple divisions of small NB-associated cells may be ongoing simultaneously in the clone and each gives rise to two daughter cells of equal size (single and double arrowheads at following intervals). **(b) **A single NB daughter cell may undergo several rounds of division. Shown are two consecutive divisions of a cell outlined with dots. Following a first symmetric division (575'–675'), the lower daughter cell underwent a second division (710'–755') while its sibling did not divide further during the recording.

The NB-like molecular expression pattern of IPs suggests that this novel type of progenitor might share some of the mitotic properties of NBs. Indeed, if the augmentation of proliferation observed in the DM lineage is mediated by amplifying IPs, these cells would be expected to divide repeatedly. To investigate this possibility, we first performed live imaging of MARCM clones on cultured brain explants dissected from third instar larvae. Clones were labeled simultaneously with CD8::GFP and tau::GFP to visualize both cell membranes and mitotic spindles (see Materials and methods). In agreement with anti-PH3 staining on fixed tissue, we observed numerous cell divisions among the small cells that were closely associated with the NB in DM NB clones (Figure 4a and Additional file 1). With the exception of the asymmetric divisions of the NB itself, all of the observed cell divisions in the clones were symmetrical (n = 75, 10 clones). Importantly, we repeatedly observed small, NB-associated cells that divided more than once. Two subsequent symmetrical divisions of such a progenitor cell are visible in the still images taken from a time-lapse laser confocal movie (Figure 4b).

**Figure 5:**
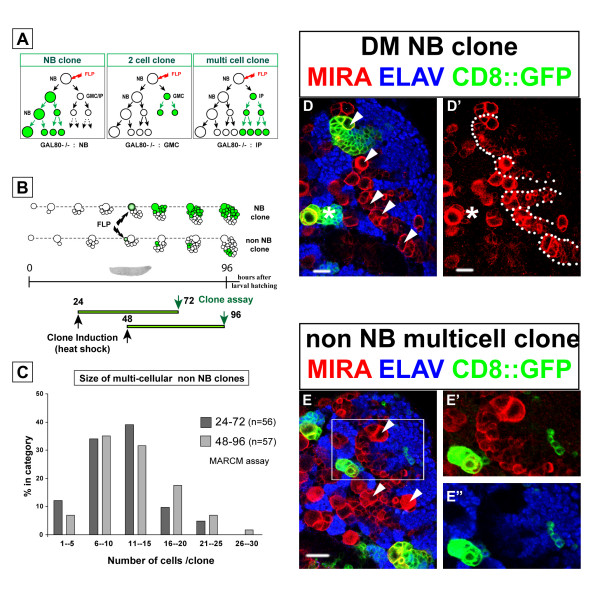
**Clonal expansion of IPs analyzed by MARCM.(a) **Schematic representation of the different types of MARCM clones that can be recovered following FLP-mediated recombination in a NB (red arrow) and segregation of homozygous GAL80 chromosomes into one of its two daughter cells (green). A multicellular clone lacking the NB (right panel) reveals the ability of the IP daughter cell to undergo several rounds of division. Not shown are FLP-mediated recombination events in the GMC or in the IP that give rise to multicellular clones only in the latter case. Recombination in the GMC gives a single labeled cell. **(b) **Top: schematic organization of multicellular GFP-labeled clones (green) after time-controlled recombination (heat-shocked FLP, black arrows) in two developing NB lineages. Bottom: unlike NB clones (upper lineage), IP clones were identified as GFP-labeled cell clusters lacking the large Miranda-positive NB and pushed away from this founder cell by proliferation (non-NB clone, lower lineage). The size and composition of clonal progenies were examined 48 hours after two independent heat-induced recombination events. **(c) **Size distribution of multicellular non-NB clones generated by recombination at 24 h (light grey bars) or 48 h (dark grey bars) ALH and assayed 48 hours later. The similar histogram profile reveals the comparable mitotic potential of progenitors present in the DM lineage at 24 or 48 h ALH. **(d, d') **Representative confocal image of NB clones induced at 48 h ALH and examined at 96 h in a dissected brain stained for the markers indicated (dorsal view, lateral to the left, anterior to the top). DM NBs are identifiable in the most medial row of large cells (arrowheads) by their association to a large cluster of Miranda-positive progenitors (various DM lineages are outlined by dots in (d); the GFP channel was omitted for clarity). The GFP-labeled progeny of a single DM NB follows the orientation of the Miranda-positive cell cluster. A typical non-DM NB clone is found on the lateral site of the brain (asterisk). This single large NB is associated with a few Miranda-positive GMCs. **(e) **Representative IP clone of four cells among the presumptive progeny of the nearest DM NB (arrowheads); same scale and conditions as in (d). A magnification of the area boxed in (e) is shown in (e', e"), with one channel omitted for clarity. The cells in the clone have undetectable level of Miranda (red) and all express the neuronal marker ELAV (blue). Scale bars: 15 μm.

Next, we performed a more detailed analysis of the different types of MARCM clones that were recoverable in the DM lineages. To date, only two types of multicellular clones have been observed in the central brain following a somatic recombination event in a parental NB and the loss of the GAL80 repressor in one of the post-mitotic siblings. Thus, the NB clones described above derive from the proliferation of GAL80-minus NB founders, while two cell clones are obtained from GAL80-minus GMCs (Figure 5a). Other possible recombination events may occur in a GMC, but they result in the labeling of a single post-mitotic daughter cell [39,41]. In DM lineages containing repeatedly dividing IPs, a third type of non-NB clone consisting of more than two labeled cells would be predicted to occur following the loss of the GAL80 repressor (Figure 5a).

Mitotic recombination was randomly induced in progenitor cells at 24 h and 48 h ALH and progenies were examined in isolated GFP-labeled clones 48 hours later (Figure 5b). As expected, single cell-, two cell-, and NB clones were recovered throughout the central nervous system. Prominent among the latter were the exceptionally large DM NB clones identifiable in the dorsal brain by their medial position and the spatial orientation of the labeled progeny that extend from the typical large cluster of late born Miranda-positive cells (Figure 5d,d'). Consistent with their linear growth rate (Figure 1d), we measured comparable clone sizes for DM NB clones generated during each of the two overlapping 48 hour windows (157 cells ± 33, n = 14 clones, and 220 cells ± 43, n = 16 clones, respectively). Likewise, non-DM NBs selected at random in the dorsal brain also generated comparable, albeit smaller, NB clones in the same time periods (63 cells ± 20, n = 40 clones, and 66 cells ± 23, n = 48 clones, respectively). Importantly, however, numerous clones lacking a NB and consisting of more than two cells were recovered in these experiments. These multicellular non-NB clones were found only in close spatial association with DM NBs and their progeny (Figure 5e,e'). Cell counts revealed a wide range of clone sizes in these lineages. Most clones, however, comprised 6–25 cells and this class was observed at comparable frequency in the two time windows examined (73% and 67%, respectively; Figure 5c). In over 90% of the cases examined, the cells in these multicellular clones expressed Elav, indicating that they were composed exclusively of post-mitotic neurons (Figure 5e,e').

The observed variability in clone size could be due to intrinsic variations in the mitotic capacity of different IPs and/or may result from mitotic recombination occurring in an IP that had already completed a variable number of divisions after its birth. Interestingly, the distribution of clonal cell number appeared remarkably similar when FLP/FRT recombination was induced at 24 h or at 48 h ALH (Figure 5c). This suggests that the mitotic potential of IPs is independent of their birth date from their parental DM NBs during larval development.

These findings imply that IPs in DM lineages can divide several times and produce differentiated progeny in less than 48 hours. Thus, they allow considerable amplification of the number of neurons produced in comparison to the standard mode of division adopted by other lineages in the central brain.

### DM neuroblasts do not segregate Prospero protein to their daughter cells

The experiments described above show that DM NBs generate multiply dividing daughter cells that produce neural progeny. Surprisingly, these amplifying IP cells appear to be restricted to the DM lineages. What might explain this restriction? DM and non-DM NBs are not morphologically distinguishable and both divide asymmetrically to generate smaller progeny cells (Figure 3 and below).

**Figure 6:**
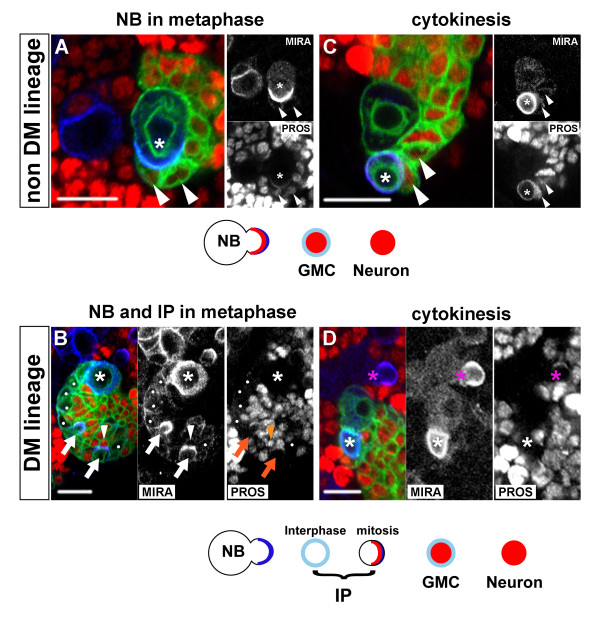
**Asymmetrically dividing DM NBs do not express Prospero.** Confocal images of NB divisions in a canonical NB lineage (top panels) compared to a DM lineage (bottom panels). Shown are representative CD8::GFP-labeled clones (green), seen around the NB in late larval brains stained for Miranda (MIRA, blue) and Prospero (PROS, red). Single channels are also shown in gray scale for better contrast. **(a, b) **Miranda forms cortical crescents at metaphase in both non-DM and DM NBs (asterisk). **(c, d) **Following asymmetric division, Miranda segregates into the small daughter cell and remains associated at high levels at the cortex soon after cytokinesis (the small newborn daughter cell is marked by an asterisk). Prospero co-localizes with Miranda in the dividing non-DM NBs (a, c, asterisks) and is nuclear in the oldest GMCs, which retain a low level of Miranda at the cortex (a, c, arrowheads), and in all other post-mitotic cells in the clone. In the DM NBs, Prospero is undetectable during mitosis (b, d, asterisks). (Note in (d) a canonical NB outside the clone (magenta asterisk) that shows co-localization of Miranda and Prospero and serves as internal control.) Recently born NB daughter cells show weak uniform cortical Miranda and lack Prospero (white dots in b). Polarized cortical Miranda during mitosis identifies these cells as IPs (b, arrows) and co-localization with Prospero is once again observed in these cells (b, insets). Cells with GMC-like (arrowheads) or neuronal expression of the markers are also observed as in canonical non-NB lineages. Scale bars: 10 μm.

A large amount of evidence indicates that the polarized assembly of multiprotein complexes at the cellular cortex during mitosis is both a characteristic hallmark of NBs and a key determinant in promoting their self-renewing ability. As exemplified in non-DM lineages (Figure 6a,c), Prospero and Miranda are synthesized in the NB and they co-localize on one side of the cortex at metaphase (Figure 6a, asterisk). This asymmetric distribution results in unequal segregation of these proteins to the budding new GMC as visualized at telophase or soon after cytokinesis (Figure 6c, asterisk). (Older GMCs located in close proximity to the newly generated GMC show a much lower level of Miranda and manifest the same type of nuclear localization of Prospero as do all other post-mitotic nuclei of the clone; Figure 6c, n > 50 clones). Importantly, the loss of these fate determinants in mosaic clones leads to unrestricted proliferation of the GMC *in situ *and the acquisition of neoplastic characters of mutant cells in transplantation assays [31-33,44].

Remarkably, and in contrast to all other *Drosophila *NBs described to date, Prospero was undetectable in the DM NBs during mitosis (Figure 6b,d). In all DM NB clones examined (n = 25), Miranda, but not Prospero, formed a cortical crescent in the dividing NB at metaphase (Figure 6b, asterisk) and segregated to the smaller daughter cell (Figure 6d). As a result, the IPs that derived directly from the DM NB lacked nuclear Prospero. GFP-labeled DM lineages typically contained 28 ± 9 Prospero-negative cells close to the NB (Figure 6b, white dots, n = 14 clones). These are likely to be accumulating IPs in interphase because they showed weak uniform expression of Miranda at the cortex and did not express PH3 (Figure 6b and data not shown). At IP mitosis, however, Prospero was unambiguously detected in these progenitors and showed co-localization with Miranda in a polarized manner (Figure 6b, arrows).

These data identify the DM NBs as a unique subset of neural stem cell-like progenitors that do not express and segregate Prospero during mitosis, thereby generating daughter cells that are molecularly distinct from GMCs.

### Intermediate progenitor cell divisions are morphologically symmetrical but molecularly asymmetrical

**Figure 7:**
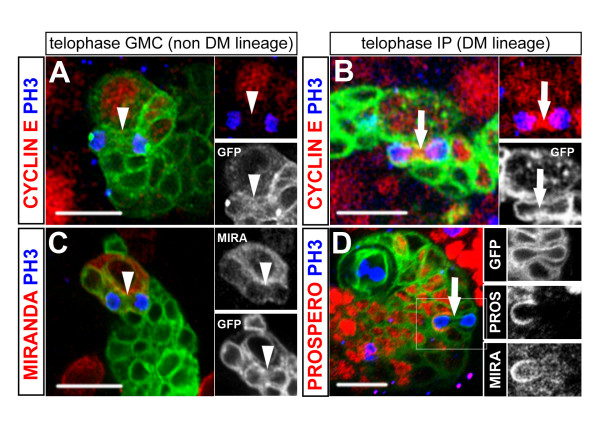
**Unequal segregation of Prospero/Miranda during symmetric division of IPs.** Confocal images of representative CD8::GFP labeled clones (green) in **(a, c) **canonical non-DM or **(b, d) **DM lineages. Shown are mitotic figures of small NB-associated cells at anaphase/telophase, visualized by anti- PH3 staining of DNA (blue). Separate channels are also shown in insets for better contrast. The outline of the plasma membrane stained by CD8::GFP shows that both the GMC (a, c, arrowheads) and the IP (b, d, arrows) divide symmetrically and give rise to daughter cells of similar sizes. The dividing IP is identified by NB-like expression of Cyclin E during mitosis (b) while GMC division lacks Cyclin E expression at this phase of the cell cycle (a). In the mitotic GMC, Miranda distributes equally to both daughter cells (c, inset) while Prospero is nuclear (see Figure 3b). In IP division, Prospero and Miranda co-segregate to only one of the two daughter cells (d, insets). Scale bars: 10 μm.

Studies on asymmetric neural stem cell division in *Drosophila *have established a simple scheme that links cell size of sibling daughter cells, restriction of mitotic potential and partitioning of fate determinants. Thus, in the canonical scheme exemplified in MARCM-labeled non-DM clones, the only self-renewing cell is the large NB that segregates Miranda/Prospero to its small GMC daughter cell during mitosis (Figure 6a,c). In contrast, the terminal division of the GMC involves the formation of equal-sized daughter cells at telophase and equal partitioning of Miranda/Prospero to both cells (n = 27; Figure 7a,c and data not shown).

The asymmetric division of DM NBs is also associated with the unequal segregation of Miranda to the smaller daughter cell (Figure 6b,d). Moreover, the resulting IP divides symmetrically to generate sibling cells of similar size as examined at telophase (n = 14; Figure 7b,d). Thus, in terms of the morphology of their cell divisions, IP cells are more like GMCs than like NBs. However, in sharp contrast to GMCs, mitotic IPs show cortical crescents of Miranda and Prospero (Figure 6b) and unequal partitioning of these two proteins at telophase (Figure 7d; n = 7). Thus, in terms of the segregation of cell fate determinants, dividing IP cells are remarkably more NB-like and differ substantially from GMCs.

Taken together, these findings demonstrate that the proliferative divisions of amplifying IPs in DM lineages have novel cellular and molecular features. These divisions are morphologically symmetrical and lead to two daughter cells of similar size, but molecularly asymmetrical in that the differentiating cell fate determinants Prospero and Miranda are segregated into only one cell. The ensuing absence of these differentiating cell fate determinants in the remaining daughter cell is likely to be a significant factor in the mitotic activity of amplifying IP cells.

## Discussion

**Figure 8:**
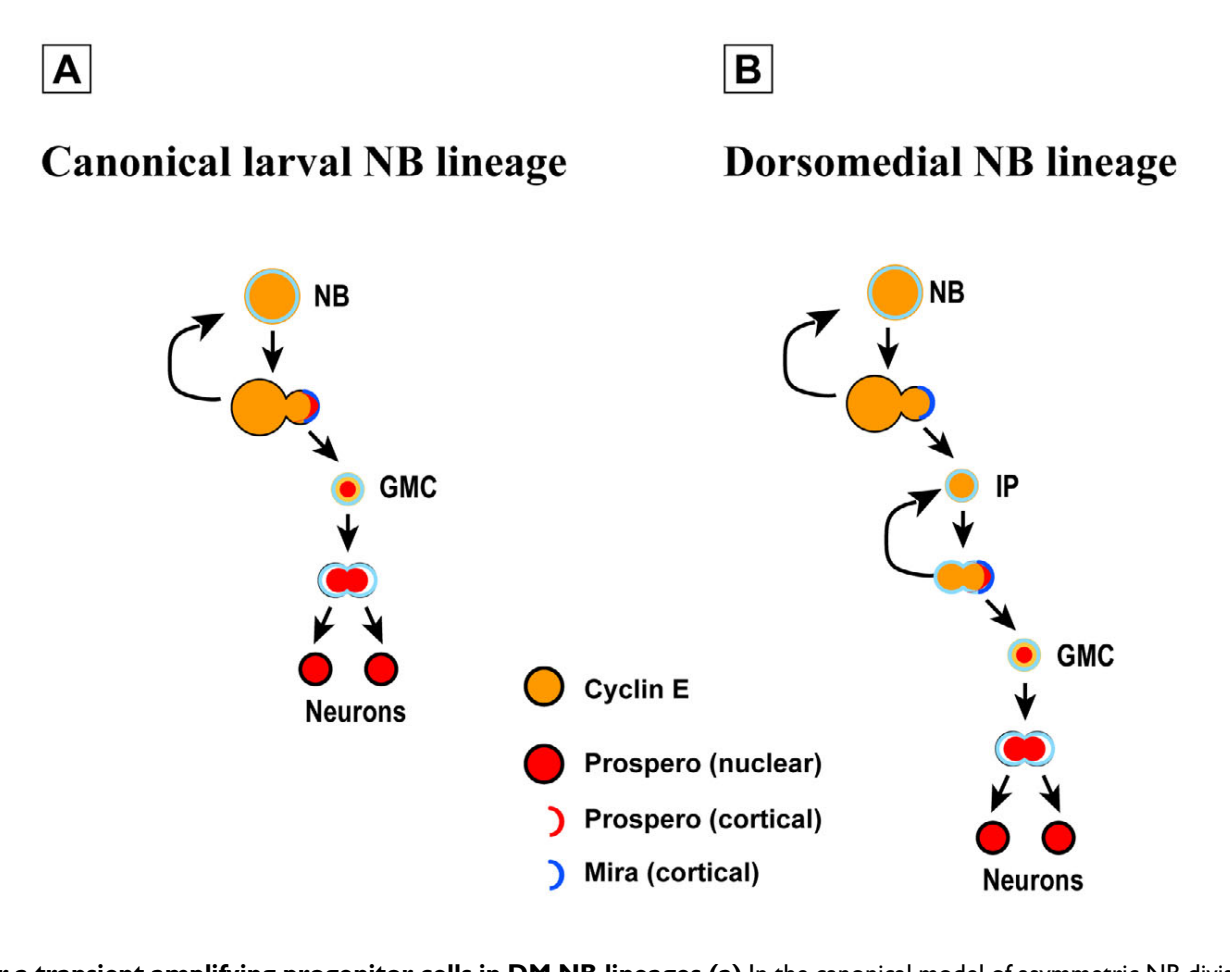
**Model for a transient amplifying progenitor cells in DM NB lineages.(a) **In the canonical model of asymmetric NB division, a single neurogenic division of the small GMC progenitor cell produces two neurons (N) at each round of NB division. Unequal partitioning of Prospero promotes neurogenic division by inhibiting self-renewing factors in the GMC. **(b) **The DM NB divides asymmetrically without Prospero, which enables the small daughter cell to retain self-renewing potential and to behave as an IP. In this cell, expression of Prospero and unknown polarization cues re-established the asymmetric segregation of fate determinants and the generation of the neurogenic progenitor GMC. This novel mode of neurogenesis increases the number of post-mitotic neurons that individual NBs in the dorsomedial brain can generate at each round of divisions.

In this report, we present cellular and molecular evidence for a new mode of neurogenesis in the larval brain of *Drosophila*. In the canonical model for postembryonic neurogenesis exemplified by the non-DM lineages of the brain and the lineages of the ventral ganglia, NBs divide asymmetrically in a stem cell mode to self-renew and generate a GMC that divides once to produce two post-mitotic cells that differentiate (Figure 8a). Associated with this process is the asymmetric segregation of the cell fate determinants Prospero and Miranda from the parent NB into the GMC, whereupon Prospero acquires a nuclear localization that is retained in the GMC's post-mitotic progeny.

The data presented here are consistent with a novel model for neurogenesis exemplified by the DM NBs, which divide asymmetrically in a stem cell mode to self-renew and generate IP daughter cells (Figure 8b). In this process, they do not segregate the cell fate determinant Prospero into the IP cells, which subsequently repeatedly divide symmetrically (in morphological aspects) yet asymmetrically segregate the cell fate determinants Prospero and Miranda during mitosis. The daughter cell that receives the Prospero and Miranda determinants is fated to become a differentiating GMC-like cell, whereas the other daughter cell retains its ability to divide several more times.

This novel model postulates that DM NBs produce exclusively IPs and not GMCs. The alternative notion, that the NB sometimes produces an IP and sometimes a GMC, is unlikely given that Prospero is never detected in the NB and, thus, cannot be segregated to one of its daughter cells as would be required for GMC generation. The model also posits that GMCs are produced by IPs through (functionally) asymmetrical divisions that result in one daughter cell becoming a GMC while the other daughter cell self-renews as an IP. Alternative scenarios, such as one in which IPs first divide symmetrically to expand in numbers and then adopt a GMC fate to generate differentiating neurons, are unlikely given the spatiotemporal pattern of Prospero/Miranda expression and the stable ratio of IPs versus GMCs observed in DM NB clones throughout larval development.

The experimental findings that support this novel model have implications for our understanding of neural stem cells and proliferation control. These are discussed in the following.

The NBs of the developing central brain and ventral ganglia divide asymmetrically in a stem cell mode in which the larger NB self renews and the smaller daughter cell differentiates into a different cell type, usually a GMC (reviewed by [7,8,10-12,18]). This asymmetric division of the parent NB has been thought to be tightly coupled with the asymmetric segregation of cell fate determinants, and central among these molecular determinants is the transcription factor Prospero, which is required in GMCs to inhibit self-renewal and to promote differentiation [20-23,27,28]. Our findings indicate that the asymmetric segregation of Prospero does not occur in all dividing brain NBs. Indeed, in the DM NBs the lack of asymmetric segregation of Prospero to the IPs may be a key element in imparting (transient) NB-like features to these proliferating cells.

The GMCs of the developing nervous system divide symmetrically and generate two postmitotic progeny of equal size. Our findings indicate that IP cells also divide symmetrically in morphological terms, although Prospero and Miranda are partitioned to only one of their daughter cells. Thus, the morphologically symmetric cell division of a NB-derived daughter cell does not necessarily engender equal portioning of differentiation factors into both resulting cells. It has been assumed that only cells of a certain critical size show NB-like proliferative properties. The small size of the GMC would be a key factor promoting cell cycle exit and differentiation of its progeny (see [8]). This simple link between cell size and self renewing/terminal division is also called into question by our findings, since IPs are comparable in size to GMCs and yet they possess a very distinct mitotic potential.

The only repeatedly dividing progenitor cell type identified to date in the central nervous system of *Drosophila *is the NB. Our studies identify the IP cell as a second progenitor type with the capacity to undergo multiple rounds of divisions. This characteristic is coupled with several cellular and molecular features that are shared with NBs. Among these are the specific expression patterns of Prospero, Miranda and CycE during mitosis as well as the ability to asymmetrically segregate Prospero and Miranda during cell division. The number of divisions that IPs typically carry out is currently not known with precision. Our observations based on quantification of cell number in multicellular clones suggest an average of three-to five divisions as a conservative estimate. If, as assumed by our model, each IP cell division results in the generation of one GMC-like daughter cell, this estimate would predict a three- to five-fold amplification of the number of neuronal progeny in DM lineages compared with other lineages of the central brain and ventral ganglia. This prediction is in reasonable accordance with the amplified cell numbers observed in NB clones of DM versus non-DM lineages. The ultimate fate of the IPs is currently not known. The fact that almost all intermediate precursor-derived multicellular clones are composed exclusively of postmitotic neurons suggests that, after multiple divisions, these cells are either eliminated by programmed cell death or that they terminally divide and differentiate.

Although the DM NBs do not express and segregate Prospero to their daughter intermediate precursors, these daughter cells do express Prospero in a cortical and polarized manner during mitosis. The off/on state of Prospero must be kept under tight control for a controlled amplification of proliferation achieved in DM lineages since complete mutational loss of Prospero in brain clones leads to uncontrolled proliferative activity and brain tumor formation [31-33,44]. Indeed, our observations on the DM lineages imply that deregulated IPs that fail to express Prospero might be an important source of tumor cells in the brain. Interestingly, region-specific action of tumor suppressor genes in the larval brain has been previously reported using somatic cell clones [32].

## Conclusion

Here we identify a novel intermediate neural progenitor generated by asymmetric division of a subset of the *Drosophila *brain neuroblasts during the postembryonic phase of neurogenesis. Unlike conventional GMC this intermediate progenitor express molecular markers of self-renewing neuroblasts and undergoes multiple divisions in absence of Prospero. In the dorsomedial brain these progenitors amplify the number of neurons that can be generated by the parental neuroblasts at each round of their divisions. The novel IP described here bears remarkable similarities to the IPs that have been identified recently in mammalian brain development (reviewed by [1-3]). In the developing mammalian brain, primary neural stem cells persist in the ventricular zone through asymmetric self-renewing divisions, and IPs, which are thought to derive from these primary neural stem cells, divide symmetrically in the adjacent subventricular zone [45-47]. The division of IPs in the subventricular zone amplifies the number of cells produced by a given neural stem cell division and may be an important determinant of brain size, since species with larger brains have a larger pool of IPs [48]. The surprising similarities in the patterns of neural stem and IP cell division in *Drosophila *and mammals suggest that amplification of brain neurogenesis in both groups of animals may rely on evolutionarily conserved cellular and molecular mechanisms.

In mammalian brain development, a two-step model has been proposed where, as a first step, asymmetric divisions of a primary neural stem cell generate a diverse set of molecularly different IPs and, as a second step, multiple symmetric divisions of each of these IPs generate large numbers of neurons of the same subtype [3]. Given this model in mammalian brain development, it will be interesting to investigate if different IPs in a given DM lineage of the *Drosophila *brain are molecularly diverse, and if the neuronal subpopulations they generate during development acquire distinguishable anatomical and functional characters. If this is the case, the expansion of neurogenesis and the generation of multiple neuronal subclasses may be intimately related in the brain development of animals as diverse as insects and mammals.

## Materials and methods

### Fly stocks and MARCM analysis

All *Drosophila *stocks were reared and maintained on standard yeast-cornmeal-agar medium and all experiments were performed at 25°C. Unless otherwise stated, fly stocks carrying transgenes and recombinant chromosomes were obtained from the Bloomington stock center and assembled using standard genetics. To generate positively marked MARCM clones y, w, hsFLP; FRT40A, tubP-GAL80^LL10^/CyO, ActGFP^JMR1^; tubP-GAL4^LL7^, UAS-mCD8::GFP^LL6^/TM6, Tb, Hu were mated to either w; FRT40A, UAS-mCD8::GFP^LL5 ^(standard cell lineage labeling with membrane-tethered GFP), or UASp-cnnGFP^26.1^; FRT40A, UAS-cLacZ^Bg4-1-2 ^(for additional labeling of centrosomes), or w; FRT40A, UAS-cLacZ^Bg4-1-2^; UAS-tauGFP^12/2/3 ^(gift of A Brand), for live imaging. Generation of MARCM clones and larval staging was performed as previously described [31] for this sub-section.

### Immunohistochemistry and live imaging

Nervous systems were dissected from larvae, fixed and immunostained as previously described [40]. Primary antibodies were as follows: rabbit anti-PH3 (1:400; Upstate, Charlottesville, Virginia, USA), mouse anti-MIRA (1:50; gift of P Overton), rabbit anti-MIRA (1:200; gift of YN Jan), mouse anti-PROS (1:10; Developmental Study Hybridoma Bank (DSHB), Iowa City, Iowa, USA); mouse anti-ELAV (1:30; DSHB) rat anti-ELAV (1:30; DSHB), mouse anti-CYCE (1:50; gift of H Richardson). Alexa Fluor-conjugated secondary antibodies (Molecular Probes, Invitrogen, Paisley, Renfrewshire, UK) were used at 1:200.

For live imaging, larval brains were dissected in Schneider's *Drosophila *Medium with 10% fetal bovine serum and mounted in 400-5 mineral oil (Sigma Diagnostic, Inc. St Louis, MO, USA) between a glass coverslip and a gas-permeable plastic foil (bioFOLIE 25, In Vitro System and Services, GmbH, Gottingen, Germany).

### Microscopy and image processing

Fluorescently stained nervous systems were imaged using a Leica TCS SP scanning confocal microscope. Z stacks were collected with optical sections at 1–1.5 μm intervals. Pictures in this paper are presented as 'thick-section' merges projected as a flat image using ImageJ [49]. Figures were assembled using Adobe Illustrator and Photoshop. Clone/lineage sizes were determined from confocal Z stacks of sections, spaced by 1 μm. Using ImageJ, cells were marked section-by-section and counted. Typically, 20–50 nervous systems per staining/genotypes/larval stages were examined using 63× oil-immersion objective. Only well isolated clones were recorded from the surface-located NB to the earliest born neurons close to the neuropil. Sample sizes, means and standard deviations for all histograms are indicated in the text and figure legends.

For time lapse, Z stacks made of 1 μm thick slices were collected at 4 minute intervals. Movies were processed and assembled using house made ImageJ plug-ins. Briefly, the sample motions were corrected in X and Y dimensions by manual reference point tracking. A single slice was arbitrarily selected per time point, allowing both some Z dimension drifting correction and the follow up of the most interesting cells within the sample.

## Competing interests

The author(s) declare that they have no competing interests.

## Authors' contributions

BB designed the study, wrote the manuscript and performed or participated in all experiments. NI performed the cell number quantification and BrdU labeling experiments. EC performed the time-lapse cell imaging experiments. HR participated in the overall design and coordination of the study and helped write the manuscript. All authors have read and approved the final manuscript.

## Supplementary Material

Additional file 1 Time-lapse analysis of a proliferating dorsomedial NB clone. Cultured explant of larval brain. CD8::GFP and tau::GFP are used to mark cell membranes and mitotic spindles simultaneously. Each frame is a projection of a thick stack of confocal sections taken at 1 μm z-intervals. Frames were taken at 1-min intervals over an 810-min period. Two divisions of the large NB cell were observed during this period (red arrowheads) while many associated cells underwent mitosis giving rise to daughter cells of similar sizes (yellow arrowheads). Click here for file
